# A bibliometric analysis of top 50-most cited articles on repetitive trans-cranial magnetic stimulation (rTMS) for treatment of depression

**DOI:** 10.1016/j.heliyon.2021.e06021

**Published:** 2021-01-26

**Authors:** Syeda Beenish Bareeqa, Syed Ijlal Ahmed, Syeda Sana Samar, Arsalan Anwar, Mustafa M. Husain

**Affiliations:** a Jinnah Medical and Dental College, Karachi, Pakistan; b Liaquat National Medical College and Hospital, Karachi, Pakistan; c Jinnah Sindh Medical University, Karachi, Pakistan; d Department of Medicine, Dow Medical College, Karachi, Pakistan; e Department of Psychiatry, UT Southwestern Medical Center, Dallas, TX, USA

**Keywords:** Citation count, Bibliometric analysis, Depression, Repetitive transcranial magnetic stimulation, Treatment

## Abstract

**Background:**

Citation count can be used as a key tool to assess the quality of the published literature and because of its immense advantages it is now widely used in ranking the articles on specific topics.

**Objective/hypothesis:**

To extract and assess the top cited work on repetitive transcranial magnetic stimulation (rTMS) for depression treatment.

**Methods:**

Scopus Library Database was searched and two independent authors produced a list of 50 most cited articles on repetitive transcranial magnetic stimulation (rTMS) for treatment of depression. All the relevant articles having key-terms within their titles, abstract and keywords were included in our search. Our list was categorized into two categories, “mixed” and “focused”.

**Results:**

The articles in the produced list of top 50 most cited articles on rTMS for treatment of depression belong to the time period 1993–2012 with total citation count 12078. George MS was prominent in the list. ‘*Biological Psychiatry*’ published most number of articles (n = 13) among the list. Articles were categorized on the basis of primary population and intervention into ‘Focused’ and ‘Mixed’ categories.

**Limitations:**

Articles that were published before 1993 and after 2012 on rTMS for depression couldn't made it to the final list of top-50 most cited article.

**Conclusion:**

We attempted to conduct a topic-specific citation analysis considering the paucity of specified bibliometrics in medical literature. Our research provides an insight on emerging trends in rTMS for depression and highlights the characteristics, quality and dynamics of frequently cited articles in the field.

## 1 Introduction

Importance of citation count has been a topic of substantial debate for some decades now. There is an inconclusive consensus on the true significance of citation rate as a quality marker for published literature (Garfield, 1979). Earlier critiques of citation counts suggested that some errors and bias devalued it as a method for assessing scholarly productivity but later analysis has suggested these errors are relatively insignificant (Meho, 2007). Reporting the citation count is important for several reasons. It helps to develop a perspective about pioneering scientific contribution within a specific field; determine the literature's impact; identify key contributors to funding agencies for prioritizing their research funds and gives a compiled version of top articles to teaching personnel (residency and fellowship program directors) which might improve academics within a field (Swindell et al., 2018).

The main role of Trans-cranial magnetic stimulation (TMS), when first developed, was its utilization as a research and diagnostic tool for various neuropsychiatric illnesses. An inadvertent finding was observed, particularly in a depressed population during research studies. Several patients reported a noticeable improvement in their depressed mood following repetitive transcranial magnetic stimulation (rTMS). This accidental but important revelation was the basis to investigate the role of rTMS as an alternative-treatment of depression. The first reported clinical use of TMS for depression was in 1987, in a study comparing mood changes after single-impulse TMS with baseline health status (Bickford et al., 1987). Depressive disorders are prevalent among the general population. It was estimated that nearly half of the affected population experiences recurrence of a depressive episode within a year (Predictable, 2006), despite being treated with antidepressant drugs, psychotherapy and electroconvulsive therapy. Appropriate pharmacotherapy with medication compliance can successfully prevent relapses however; antidepressant drugs have few troublesome side effects if prescribed for an extended period of time (Predictable, 2006). Therefore, the discovery of rTMS as a therapeutic modality is considered a significant alternative for treatment-resistant depression (Rachid, 2018).

Bibliometric analysis has been conducted on numerous clinical and diagnostic aspects in neuropsychiatry including depression (Lipsman and Lozano, 2011), selective serotonin reuptake inhibitors (López-Muñoz et al., 2003), neuroimaging (Kim et al., 2016) and classification/diagnosis of psychiatric illnesses (López-Muñoz et al., 2008). However, after an extensive search of published literature, only one citation analysis was found on biological treatment of depression that discussed rTMS metrics. Recently, a group of researchers conducted a scientometric analysis on biological treatments for Major Depressive Disorder (MDD) (Bach X Tran et al., 2019). The popularity index (PI) is the share of publications on a specific topic (the name of an antidepressant or neuro-stimulation therapy) relative to all articles in the field of MDD from 1988 to 2017. The PI of electro-convulsive therapy (ECT) was highest in the field of Psychiatry (2.55). The PIs of rTMS was 1.04 and ranked second and it was higher than PI of vagus nerve stimulation (0.23), deep brain stimulation (0.56) and transcranial direct current stimulation (0.29).

In this study, we aim to map the trends of research on rTMS for depression during period of 1993–2017, which would guide scholars to realize the panorama of rTMS for depression-related research, and define the future research direction.

## 2 Methodology

The top 50-most cited published articles on repetitive transcranial magnetic stimulation (rTMS) for treatment of depression were identified in October 2019 using the Scopus Library Database (www.scopus.com). All articles containing the following terms like “Transcranial Magnetic Stimulation” OR “Repetitive Transcranial Magnetic Stimulation” AND “Depression” within their titles, abstract and keywords were included in our search. The search strategy was extended using keywords like “Treatment Resistant Depression” OR “Depressed” OR “Depressive” OR “Major Depressive Disorder” AND “Transcranial magnetic stimulation” in abstracts and main texts. Details of Scopus search is given in Supplementary file 1. The literature search was limited to original and review articles whereas conference papers, letters, editorials, commentaries, and book references were excluded from our study. We selected ‘Journals’ only in the source type section for the selection of articles. No limitations based on time period, type of review/original article, funded or non-funded research and origin of the article were applied to this analysis.

Articles were included only if the primary intervention was repetitive transcranial magnetic stimulation for treating any type of depression (e.g. Major depressive disorder, atypical depression, bipolar depression etc). Whereas, those article were excluded where primary intervention was not rTMS (e.g. single pulse TMS or paired pulse TMS) or the condition treated was other than depression (e.g. Parkinson's disease).

After an extensive search, the initial list of relevant articles was sorted under the category of “Cited by highest” and two reviewers (SBB & SSS) independently generated the preliminary lists of top 50-most cited articles on rTMS for depression. Two lists were compared and detailed screening of abstracts and full texts was performed to avoid the selection bias and to analyze the accuracy of the search strategy. A discrepancy of 7% was found between the two preliminary lists, which was resolved by discussion with the third independent author (SIA) and with a collaborative consensus of all reviewers to generate a final list.

The list was generally categorized into ‘Focused’ and ‘Mixed’ articles. The focused category comprised articles in which the primary intervention and population was rTMS and depression respectively. However, the mixed category was composed of such studies that assessed an additional primary intervention within an additional population apart from rTMS and depression. These general categories were sub-classified on the basis of study design, which included randomized controlled trials (RCTs), cohorts, case report/series, pilot studies, systematic review/meta-analysis (sys/meta) and others. For each study selected in the top-50 list, relevant data including citation count, top authors with their affiliation, year of publication, type of studies, the top-journals name with their recent impact factors and country of origin was tabulated. The articles were ranked on the basis of a total number of citations. Additionally, calculation of citation density was done to identify the corrected ranks for each article. Citation density is citation count per year, which is obtained by dividing the total number of citations to the number of years it has been since the publication date.

Periodical charting of a total number of articles along with the mean number of citations by 5-year interval was done for the timeline of 1993–2017. Moreover, using statistics extracted via Scopus search, studies were charted out on the basis of country of origin. Journals with 3 or more articles within the top-50 list were included in the top-sources list. Authors with 4 or more publications in top-50 articles were selected as leading authors for ‘role of rTMS in depression’ related research. These quantitative indicators will provide a snippet on bibliometric trends, focus and shortcomings in rTMS for depression-related research and it will define the future goals (direction of funding) to improve the chances of development within the field.

Pearson product-moment correlation coefficient test was used to analyze the linear relation between the journal's impact factor and the number of articles from the top-50 list. A p-value of <0.05 was considered significant for all the statistical analysis. Data is primarily presented in mean, median and inter-quartile ranges.

## 3 Results

The total number of articles recovered were 12,108. After excluding the conference papers, letters, editorials, commentaries, and book references, the number of remaining articles was 11,791. Afterwards, two independent reviewers screened the title and abstract to include articles on the basis of predefined inclusion criteria. They recovered the list of 6881 relevant articles which was arranged by using ‘Cited by highest’ option. After arranging the shortlisted articles in the order of decreasing number of citation count, two independent reviewer were asked to review full texts of articles in consecutive manner till they generate the list of top-50 articles on rTMS for depression.

The top-50 highest cited articles on repetitive transcranial magnetic stimulation for the treatment of depression were published during the time period 1993–2012 with citation count ranging from 830 to 124. The total citation count was 12078 with the mean of 241.56, median of 533 and inter-quartile range of ±98.75. The average citation density ranged from 6.25 to 69 citations per year. Corrected ranks on the basis of citation density were ranging from -22 to +21. Detail of article, original rank, total citation count, the average number of citations per year, and corrected rank is given in supplementary table (Table S2).

There were 31 articles which received funding for their studies. Additionally, when extracted articles were divided into ‘Focused’ and ‘Mixed’ category, it was found that 76% articles in top-50 list were focused and only 24% articles has fallen into mixed category.

**Figure 1:**
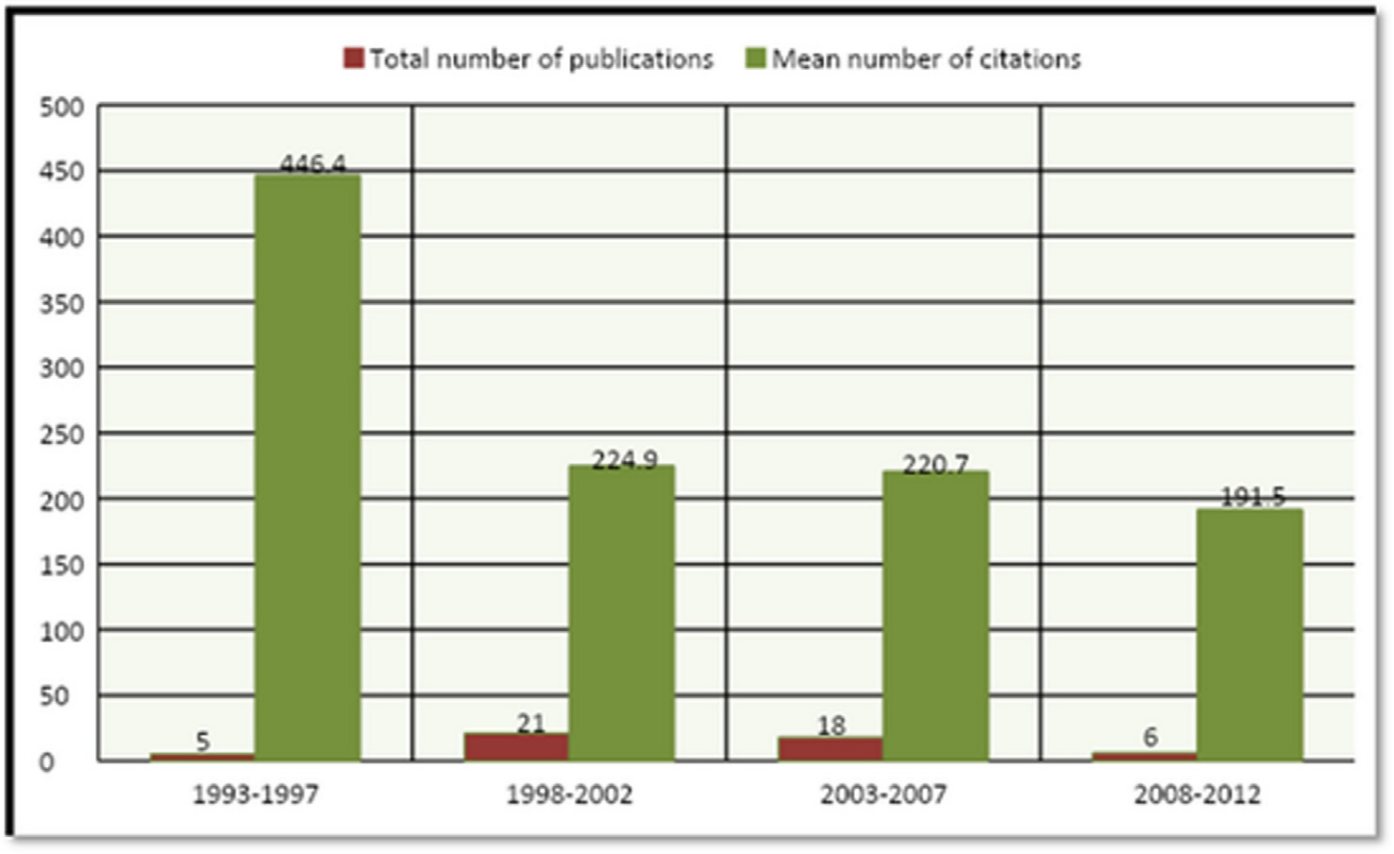
Distribution of total number of publications and mean number of citations on the basis of 5-year interval.

Most numbers of articles (n = 21) were published during the 5-year period of 1998–2002 however, the highest mean number of citations (n = 446.4) was from 1993 to 1997. Details of the number of publications in comparison with mean citation count by 5-year interval are presented in Figure 1.

Total of 160 authors contributed in top-50 articles out of which, only 11 authors had 4 or more publications within the list. Mark George was outstanding in the list with 9 publications on rTMS for treating depression. The number of articles by an author was ranging from 1 to 9 (median = 1) articles. Top-authors detail is given in Table 1.

**Table 1:** Top scholars in articles of rTMS for treating depression.

Author Name	Total Number of Articles in list	Author Position in the article			Affiliation(s)
		First	Last	Other	
Mark George	9	3	4	2	Medical University of South Carolina, Charleston, United States
Nahas Z	5	1	0	4	University of Minnesota, Department of Psychiatry, Minneapolis, Unied States.
Wassermann EM	4	0	0	4	Universita degli Studi di Genova, Department of Neurology, Genova, Italy
Speer AM	4	1	0	3	National Institute of Mental Health, Bethesda, United States
Sackeim HA	4	0	3	1	Columbia University, Department of Psychiatry and Radiology New York, United States
Pascaul LA	4	1	1	2	Harvard Medical School Boston, United States
Lisanby SH	4	2	0	2	National Institute of Mental Health, Bethesda, United States
Grunhaus L	4	2	1	1	Jerusalem Mental Health Center-Ezrath Nashim, Jerusalem, Israel
Fitzgerald PB	4	3	0	1	Epworth HealthCare, Melbourne, Australia
Fregni F	4	2	1	1	Harvard Medical School, Boston, United States
Daskalakis ZJ	4	0	2	2	Centre for Addiction and Mental Health, Toronto, Canada

Similarly, articles in top-50 lists were published in 23 different journals. Total of 5 journals had 3 or more publications within the top-50 list with ‘Biological Psychiatry’ being the most prominent source (n = 13). Journals included in the top-sources list had more than half of the contribution (56%) in top-50 articles on rTMS for depression. Range of articles published by each journal was varying from 1 to 13 (median = 1) publications. Tabulated data on top-sources is presented in Table 2. There was evidence of significant (p=<0.05) association between the journal's impact factor and the number of top-50 cited articles.

**Table 2:** Top sources with most articles in top-50 list.

Journal's Name	Number of Articles	Impact Factor 2017
Biological Psychiatry	13	11.982
American Journal of Psychiatry	5	13.391
Neuropsychopharmacology	4	6.544
International Journal Of Neuropsychopharmacology	3	3.981
Journal of Neuropsychiatry And Clinical Neurosciences	3	1.854

**Figure 2:**
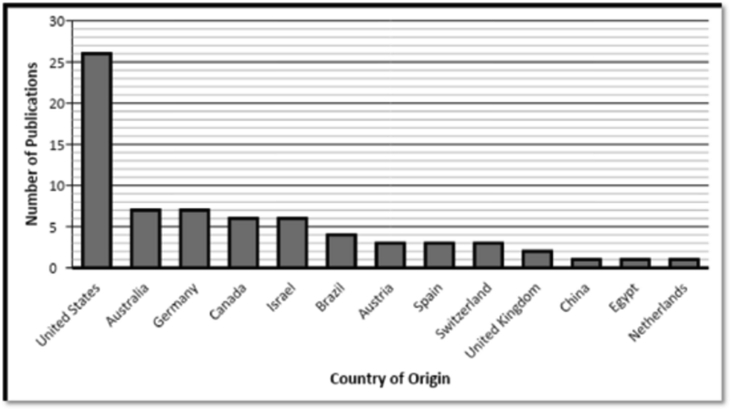
Distribution of articles on the basis of country of origin.

**Figure 3:**
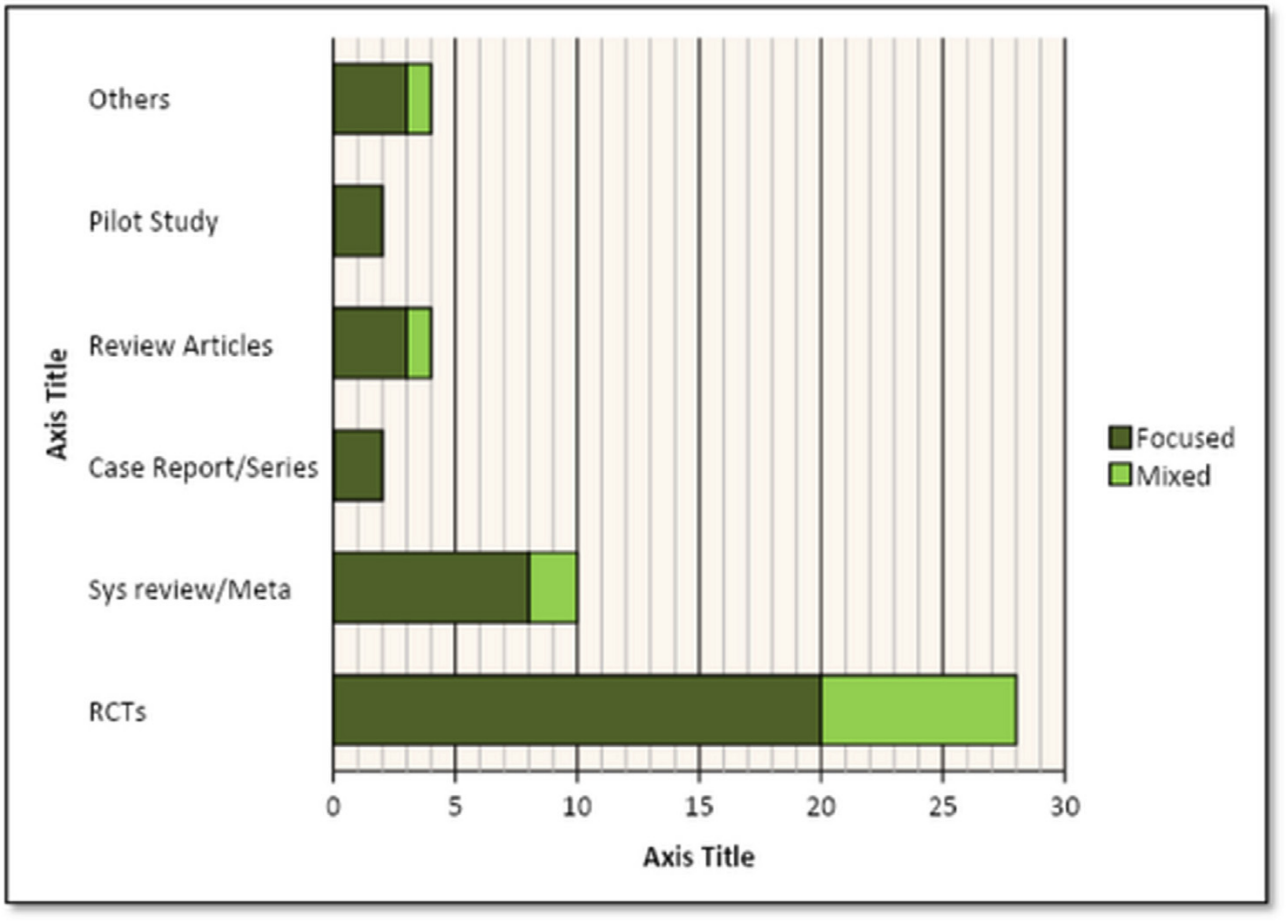
Type of article with sub-division of study types.

Articles in the top-50 list originated from 13 different countries. Most numbers of highly cited articles on rTMS for depression belonged to the United States (n = 26) followed by Australia (n = 7) and Germany (n = 7). Figure 2 demonstrated the country-based allocation of articles. Articles were distributed on the basis of primary population and intervention into ‘Focused’ and ‘Mixed’ categories. Focused-articles had 38 studies including 20 randomized controlled trials (RCTs), 8 systematic reviews and meta-analysis (Sys-review/Meta), 3 review articles, 3 other articles, 2 case reports/series, and 2 pilot studies. However, the mixed-articles category had 8 RCTs, 2 Sys-review/Meta, 1 review and 1 other article. Flow diagram of article distribution is presented in Figure 3.

## 4 Discussion

Growth of scientific research on depression has always been a point of great interest for psychiatrists. Trends in different clinical aspects such as diagnosis and treatment of depression have been evolving rapidly. Which is why, funding agencies, both private and federal corporate, have shown interest in this field. From our search on Scopus, we observed that almost two-third of the articles in the top-50 most cited list received funding for their study. Articles funded by private agencies dominated the list as compared to the researches that received funding from state corporations. However, no positive association between funding and citation rate was observed. Our results correlate with other researches where authors demonstrated that the scientific impact of funding has no significant influence on bibliometrics of specific research (Fortin and Currie, 2013). The primary reason behind growing interest of funding agencies in depression-related research is the increasing prevalence of depression around the globe. According to the world health organization (WHO) report on the global burden of disease, depression solely is the most significant contributor to worldwide disability. Nearly 4.4% people around the world suffer from this debilitating illness. Among several serious outcomes of untreated or refractory depression, suicide has been the most serious outcome, with an estimated rate as high as 800,000 people per annum (World Health, 2017). A bibliometric analysis on neuro-stimulatory intervention for depression will guide funding agencies in effective allocation of resources, identify the suitable researches, institutes and countries for collaboration and focusing on the unexplored dimensions within a field.

We believe that specificity of a topic in bibliometric analysis is essential, which is generally an unpopular trend in bibliometrics as observed. Articles which cover wider or multiple domains are naturally more likely to get cited as compared to articles related to a particular theme-centered topic. This could project a false sense of significance about a study to the clinicians within the particular field, even if the study is not entirely relevant to the clinician's sub-specialty. This pattern can be observed by comparing two different bibliometric studies. Among the list of top-100 most pain & depression articles (a non-specific topic); topmost article in the list had 1576 citation (Du et al., 2019) which is far greater than the highest number of citation per year (total 934 citations) on the top 55 articles in 2015 related to artificial intelligence in managing MDD (comparatively a specific topic) (Bach Xuan Tran, Roger S McIntyre, et al., 2019). Therefore, considering the paucity of topic-specific bibliometric analysis, we've analyzed the metrics of rTMS for treating depression which is relatively a specific topic.

Treating depression can be an intricate process due to its unpredictable prognosis and multiple inciting factors. Treatment modalities for depression are numerous. Some commonly used treatment options are cognitive behavioral therapy, selective serotonin reuptake inhibitors, serotonin-norepinephrine reuptake inhibitors, atypical antidepressants, electroconvulsive therapy, and repetitive transcranial magnetic stimulation (Thase et al., 1997). Therefore, the task of extracting data particularly specific to our topic was challenging. According to our findings, there was a significant difference observed between the metrics of ‘focused’ versus ‘mixed’ articles (p=<0.05). ‘Focused’ articles contributed more to the top-50 most cited articles list as compared to ‘Mixed’ articles. In contrast to our research, topics with a broader spectrum such as spontaneous intracerebral hemorrhage show a greater percentage of non-specific articles (52%) as compared to focused articles (Nasir et al., 2018). We can postulate from this finding that specification of a topic with the comprehensive search using different keywords can produce focused results while conducting bibliometric analysis. This might help physicians to identify leading researches for a certain subject in their specialty.

Regarding study type, our results showed that randomized controlled trial (56%) was a leading form of research among all other types for rTMS and depression related research. A similar trend of RCTs being frequently cited as a type of study can be observed for acupuncture research as well (Ma et al., 2016). According to our analysis, the United States (n = 26) has contributed most in the field of rTMS for depression. This finding is common and comparable to other bibliometric analysis like on Parkinson's disease (Li et al., 2008), Diabetes (Krishnamoorthy et al., 2009) and obstructive sleep apnea research (Huang, 2009).

### 4.1 Limitations

There are several limitations that need to be considered in our present study. No article that was published before 1993 and after 2012 on rTMS for depression, made it to the final list of top-50 most cited articles. Reason for this spontaneous exclusion of recently published articles is due to ‘citation bias’ associated with bibliometric analysis (Seglen, 1998). This resulted in the exclusion of possibly high-impact research with insufficient citation count to make it to the final list. In this way, one can also miss out on such latest studies which gives an insight about groundbreaking advancements in the field. Additionally, the possibility of missing articles older than 1993 could have been due to the fact that online databases had limitations in tracking older articles. However, to fulfill the purpose of providing detailed insight into leading researchers in the field of rTMS for treating depression, 50 articles were felt both appropriate and sufficient. Secondly, we extracted articles published in the English language which might have omitted some potentially impactful articles in other languages. Furthermore, we haven't considered ‘self-citation’ that could be an influencing factor for total citation count. Previous bibliometric analysis in mental health and medicine create visualization graphs indicating contributions and collaborative efforts of different countries on a particular research topic (Bach Xuan Tran, Mackenzie Moir, et al., 2019; Bach Xuan Tran, Giang Thu Vu, et al., 2019). This study did not explore collaboration between different countries. Lastly, there is a slight possibility to miss out such prominent researches that used key terms other than the terms utilized in our search strategy. Since we utilized only a single online database for our analysis, there is a possibility that our results show some disparity if performed on a different electronic engine like Google Scholar or Web of Science.

## 5 Conclusion

In conclusion, our study recognizes the trends noticed in scientific publications in the field of rTMS for treating depression, which may benefit the authors, reviewers and editors of journals in directing their efforts to bring about most prominent scholarly work in terms of readership, and also aid in prioritizing research funding. After conducting a quantitative analysis of the top-50 most cited articles, it was observed that high impact articles are usually published in field-specific journals, have been published from USA, are usually authored by a pool of eminent Psychiatrist and are published between 1993 and 2012.

## Declarations

### Author contribution statement

Syeda Beenish Bareeqa: Conceived and designed the experiments; Performed the experiments; Analyzed and interpreted the data; Contributed reagents, materials, analysis tools or data; Wrote the paper.

Syed Ijlal Ahmed: Performed the experiments; Analyzed and interpreted the data; Contributed reagents, materials, analysis tools or data; Wrote the paper.

Syeda Sana Samar: Performed the experiments; Analyzed and interpreted the data; Wrote the paper.

Arsalan Anwar: Analyzed and interpreted the data; Contributed reagents, materials, analysis tools or data.

Mustafa M. Husain: Conceived and designed the experiments; Wrote the paper.

### Funding statement

This research did not receive any specific grant from funding agencies in the public, commercial, or not-for-profit sectors.

### Data availability statement

Data will be made available on request.

### Declaration of interests statement

The authors declare no conflict of interest.

### Additional information

No additional information is available for this paper.
