# Supplementary material for: A bibliometric analysis of top 50-most cited articles on repetitive trans-cranial magnetic stimulation (rTMS) for treatment of depression
Source: Heliyon. 2021 Jan 26;7(1):e06021. doi: 10.1016/j.heliyon.2021.e06021 (PMC7841314; doi:10.1016/j.heliyon.2021.e06021)
Supplement: Supplementary 1 [file mmc1.docx]

**SEARCH STRATEGY IN SCOPUS**

( ( ( ( TITLE ( *Depress* OR *Major depressive disorder* OR "Affective disorder*" OR "Atypical depression*" OR "Treatment-resistant depression*" ) AND ( *repetitive transcranial magnetic stimulation* OR *rTMS* OR "transcranial magnetic stimulation*") ) OR KEY ( ( *Depress* OR *Major depressive disorder* OR "Affective disorder*" OR "Atypical depression*" OR "Treatment-resistant depression*" ) AND ( *repetitive transcranial magnetic stimulation* OR *rTMS* OR "transcranial magnetic stimulation*" ) ) OR ( ( INDEXTERMS ( "Depressed*" OR "Depressive*" OR "Drug resistant depression*" OR "Mood disorder*" OR "Anti-depressant resistant depression" OR “Unipolar depression” OR "Bipolar depression" OR "Anhedonia" OR "Flat affect" OR “Fatigue” OR "Feeling of guilt" OR "Insomnia*" OR “Hypersomnia” OR “Change in apetitie” OR "Loss of appetite*" OR "Loss of interest*" OR “Suicide” OR "Suicidal ideation*" OR "suicide *" OR "Sad mood*" OR "Low energy*" OR “Impaired concentration” OR "functional impairment*" OR “Psychomotor retardation” OR "Patient health questionnaire scale" OR "PHQ-9" OR "DSM-V" OR "DSM-IV" OR "Geriatric depression scale" OR "Hamilton depression rating scale" ) ) AND ( ( "Magnetic stimulation*" OR "Repetitive stimulation" OR "Transient magnetic field stimulation" OR "Direct brain stimulation therapies" OR "antidepressant therapies*" OR "Electrophysiologic intervention" OR "Biological intervention" OR "rTMS for depression") ) OR ABS ( (*Depress* OR *Major depressive disorder* OR "Affective disorder*" OR "Atypical depression*" OR "Treatment-resistant depression*" ) AND ( *repetitive transcranial magnetic stimulation* OR *rTMS* OR "transcranial magnetic stimulation*" ) ) ) ) AND NOT ( ( TITLE-ABS-KEY ( *Single-pulse transcranial magnetic stimulation* OR paired pulse transcranial magnetic stimulation* ) )
