# Supplementary material for: A bibliometric analysis of top 50-most cited articles on repetitive trans-cranial magnetic stimulation (rTMS) for treatment of depression
Source: Heliyon. 2021 Jan 26;7(1):e06021. doi: 10.1016/j.heliyon.2021.e06021 (PMC7841314; doi:10.1016/j.heliyon.2021.e06021)
Supplement: Supplementary 2 [file mmc2.docx]

Table S2: List of top-50 most cited articles on rTMS for depression.

| Rank | Article Detail | Number of Citations | Average Number of Citations per Year | Corrected Rank |
| --- | --- | --- | --- | --- |
|  | Pascual-Leone A, Rubio B, PallardÃ³ F, CatalÃ¡ MD. Rapid-rate transcranial magnetic stimulation of left dorsolateral prefrontal cortex in drug-resistant depression. Lancet. 1996;348(9022):233-7. | 830 | 37.00 | 2 |
|  | O'Reardon JP, Solvason HB, Janicak PG, Sampson S, Isenberg KE, Nahas Z, et al. Efficacy and Safety of Transcranial Magnetic Stimulation in the Acute Treatment of Major Depression: A Multisite Randomized Controlled Trial. Biological Psychiatry. 2007;62(11):1208-16. | 759 | 69 | 1 |
|  | George MS, Wassermann EM, Williams WA, Callahan A, Ketter TA, Basser P, et al. Daily repetitive transcranial magnetic stimulation (rTMS) improves mood in depression. NeuroReport. 1995;6(14):1853-6. | 619 | 26.9 | 4 |
|  | Klein E, Kreinin I, Chistyakov A, Koren D, Mecz L, Marmur S, et al. Therapeutic efficacy of right prefrontal slow repetitive transcranial magnetic stimulation in major depression: A double-blind controlled study. Archives of General Psychiatry. 1999;56(4):315-20. | 447 | 23.5 | 8 |
|  | George MS, Wassermann EM, Kimbrell TA, Little JT, Williams WE, Danielson AL, et al. Mood improvement following daily left prefrontal repetitive transcranial magnetic stimulation in patients with depression: A placebo-controlled crossover trial. American Journal of Psychiatry. 1997;154(12):1752-6. | 432 | 20.57 | 10 |
|  | Gershon AA, Dannon PN, Grunhaus L. Transcranial magnetic stimulation in the treatment of depression. American Journal of Psychiatry. 2003;160(5):835-45. | 408 | 27.20 | 3 |
|  | Burt T, Lisanby SH, Sackeim HA. Neuropsychiatric applications of transcranial magnetic stimulation: A meta-analysis. International Journal of Neuropsychopharmacology. 2002;5(1):73-103. | 397 | 24.81 | 7 |
|  | Speer AM, Kimbrell TA, Wassermann EM, Repella JD, Willis MW, Herscovitch P, et al. Opposite effects of high and low frequency rTMS on regional brain activity in depressed patients. Biological Psychiatry. 2000;48(12):1133-41. | 394 | 21.88 | 9 |
|  | George MS, Nahas Z, Molloy M, Speer AM, Oliver NC, Li XB, et al. A controlled trial of daily left prefrontal cortex TMS for treating depression. Biological Psychiatry. 2000;48(10):962-70. | 314 | 17.44 | 14 |
|  | Grunhaus L, Dannon PN, Schreiber S, Dolberg OH, Amiaz R, Ziv R, et al. Repetitive transcranial magnetic stimulation is as effective as electroconvulsive therapy in the treatment of nondelusional major depressive disorder: An open study. Biological Psychiatry. 2000;47(4):314- | 275 | 15.22 | 20 |
|  | Grimm S, Beck J, Schuepbach D, Hell D, Boesiger P, Bermpohl F, et al. Imbalance between Left and Right Dorsolateral Prefrontal Cortex in Major Depression Is Linked to Negative Emotional Judgment: An fMRI Study in Severe Major Depressive Disorder. Biological Psychiatry. 2008;63(4):369-76. | 263 | 26.30 | 6 |
|  | Martin OLR, Barbanoj MJ, Schlaepfer TE, Thompson E, PÃ©rez V, Kulisevsky J. Repetitive transcranial magnetic stimulation for the treatment of depression: Systematic review and meta-analysis. British Journal of Psychiatry. 2003;182(JUNE):480-91. | 259 | 17.22 | 16 |
|  | Kimbrell TA, Little JT, Dunn RT, Frye MA, Greenberg BD, Wassermann EM, et al. Frequency dependence of antidepressant response to left prefrontal repetitive transcranial magnetic stimulation (rTMS) as a function of baseline cerebral glucose metabolism. Biological Psychiatry. 1999;46(12):1603-13. | 257 | 13.52 | 23 |
|  | Schutter DJLG. Antidepressant efficacy of high-frequency transcranial magnetic stimulation over the left dorsolateral prefrontal cortex in double-blind sham-controlled designs: A meta-analysis. Psychological Medicine. 2009;39(1):65-75. | 241 | 26.77 | 5 |
|  | Berman RM, Narasimhan M, Sanacora G, Miano AP, Hoffman RE, Hu XS, et al. A randomized clinical trial of repetitive transcranial magnetic stimulation in the treatment of major depression. Biological Psychiatry. 2000;47(4):332-7. | 234 | 13.00 | 27 |
|  | Fitzgerald PB, Benitez J, De Castella A, Daskalakis ZJ, Brown TL, Kulkarni J. A randomized, controlled trial of sequential bilateral repetitive transcranial magnetic stimulation for treatment-resistant depression. American Journal of Psychiatry. 2006;163(1):88-94. | 232 | 19.33 | 11 |
|  | Padberg F, Zwanzger P, Keck ME, Kathmann N, Mikhaiel P, Ella R, et al. Repetitive transcranial magnetic stimulation (rTMS) in major depression: Relation between efficacy and stimulation intensity. Neuropsychopharmacology. 2002;27(4):638-45. | 230 | 12.10 | 29 |
|  | Kozel FA, Nahas Z, DeBrux C, Molloy M, Lorberbaum JP, Bohning D, et al. How coil-cortex distance relates to age, motor threshold, and antidepressant response to repetitive transcranial magnetic stimulation. Journal of Neuropsychiatry and Clinical Neurosciences. 2000;12(3):376-84. | 223 | 12.388 | 26 |
|  | Loo C, Mitchell P, Sachdev P, McDarmont B, Parker G, Gandevia S. Double-blind controlled investigation of transcranial magnetic stimulation for the treatment of resistant major depression. American Journal of Psychiatry. 1999;156(6):946-8. | 221 | 11.63 | 32 |
|  | Avery DH, Holtzheimer Iii PE, Fawaz W, Russo J, Neumaier J, Dunner DL, et al. A controlled study of repetitive transcranial magnetic stimulation in medication-resistant major depression. Biological Psychiatry. 2006;59(2):187-94. | 210 | 17.50 | 15 |
|  | Grunhaus L, Schreiber S, Dolberg OT, Polak D, Dannon PN. A randomized controlled comparison of electroconvulsive therapy and repetitive transcranial magnetic stimulation in severe and resistant nonpsychotic major depression. Biological Psychiatry. 2003;53(4):324-31 | 207 | 13.80 | 22 |
|  | Höflich G, Kasper S, Hufnagel A, Ruhrmann S, Möller HJ. Application of transcranial magnetic stimulation in treatment of drug‐resistant major depression—a report of two cases. Human Psychopharmacology: Clinical and Experimental. 1993;8(5):361-5. | 199 | 15.30 | 19 |
|  | Figiel GS, Epstein C, McDonald WM, Amazon-Leece J, Figiel L, Saldivia A, et al. The use of rapid-rate transcranial magnetic stimulation (rTMS) in refractory depressed patients. Journal of Neuropsychiatry and Clinical Neurosciences. 1998;10(1):20-5. | 197 | 9.85 | 40 |
|  | HÃ¶flich G, Kasper S, Hufnagel A, Ruhrmann S, MÃ¶ller HâJ. Application of transcranial magnetic stimulation in treatment of drugâ€resistant major depressionâ€”a report of two cases. Human Psychopharmacology: Clinical and Experimental. 1993;8(5):361-5. | 196 | 7.84 | 46 |
|  | Gross M, Nakamura L, Pascual-Leone A, Fregni F. Has repetitive transcranial magnetic stimulation (rTMS) treatment for depression improved? A systematic review and meta-analysis comparing the recent vs. the earlier rTMS studies. Acta Psychiatrica Scandinavica. 2007;116(3):165-73. | 185 | 16.81 | 17 |
|  | Holtzheimer 3rd PE, Russo J, Avery DH. A meta-analysis of repetitive transcranial magnetic stimulation in the treatment of depression. Psychopharmacology bulletin. 2001;35(4):149-69. | 184 | 10.82 | 37 |
|  | Teneback CC, Nahas Z, Speer AM, Molloy M, Stallings LE, Spicer KM, et al. Changes in prefrontal cortex and paralimbic activity in depression following two weeks of daily left prefrontal TMS. Journal of Neuropsychiatry and Clinical Neurosciences. 1999;11(4):426-35. | 183 | 9.63 | 41 |
|  | McNamara B, Ray JL, Arthurs OJ, Boniface S. Transcranial magnetic stimulation for depression and other psychiatric disorders. Psychological Medicine. 2001;31(7):1141-6. | 180 | 10.58 | 38 |
|  | Lisanby SH, Luber B, Schlaepfer TE, Sackeim HA. Safety and feasibility of magnetic seizure therapy (MST) in major depression: Randomized within-subject comparison with electroconvulsive therapy. Neuropsychopharmacology. 2003;28(10):1852-65. | 179 | 11.93 | 30 |
|  | Lam RW, Chan P, Wilkins-Ho M, Yatham LN. Repetitive transcranial magnetic stimulation for treatment-resistant depression: A systematic review and metaanalysis. Canadian Journal of Psychiatry. 2008;53(9):621-31. | 178 | 17.80 | 13 |
|  | Janicak PG, Dowd SM, Martis B, Alam D, Beedle D, Krasuski J, et al. Repetitive transcranial magnetic stimulation versus electroconvulsive therapy for major depression: Preliminary results of a randomized trial. Biological Psychiatry. 2002;51(8):659-67. | 169 | 10.56 | 39 |
|  | Lisanby SH, Husain MM, Rosenquist PB, Maixner D, Gutierrez R, Krystal A, et al. Daily left prefrontal repetitive transcranial magnetic stimulation in the acute treatment of major depression: Clinical predictors of outcome in a multisite, randomized controlled clinical trial. | 168 | 18.66 | 12 |
|  | Nahas Z, Kozel FA, Li X, Anderson B, George MS. Left prefrontal transcranial magnetic stimulation (TMS) treatment of depression in bipolar affective disorder: A pilot study of acute safety and efficacy. Bipolar Disorders. 2003;5(1):40-7. | 164 | 10.93 | 36 |
|  | Fregni F, Santos CM, Myczkowski ML, Rigolino R, Gallucci-Neto J, Barbosa ER, et al. Repetitive transcranial magnetic stimulation is as effective as fluoxetine in the treatment of depression in patients with Parkinson's disease. Journal of Neurology, Neurosurgery and Psychiatry. 2004;75(8):1171-4. | 163 | 11.64 | 31 |
|  | Fitzgerald PB, Oxley TJ, Laird AR, Kulkarni J, Egan GF, Daskalakis ZJ. An analysis of functional neuroimaging studies of dorsolateral prefrontal cortical activity in depression. Psychiatry Research - Neuroimaging. 2006;148(1):33-45. | 161 | 13.41 | 24 |
|  | Couturier JL. Efficacy of rapid-rate repetitive transcranial magnetic stimulation in the treatment of depression: A systematic review and meta-analysis. Journal of Psychiatry and Neuroscience. 2005;30(2):83-90. | 161 | 12.384 | 28 |
|  | Kolbinger HM, HÃ¶flich G, Hufnagel A, MÃ¼ller HâJ, Kasper S. Transcranial magnetic stimulation (TMS) in the treatment of major depression â€” a pilot study. Human Psychopharmacology: Clinical and Experimental. 1995;10(4):305-10. | 155 | 6.73 | 49 |
|  | Triggs WJ, McCoy KJM, Greer R, Rossi F, Bowers D, Kortenkamp S, et al. Effects of left frontal transcranial magnetic stimulation on depressed mood, cognition, and corticomotor threshold. Biological Psychiatry. 1999;45(11):1440-6. | 154 | 8.10 | 45 |
|  | Fitzgerald PB, Hoy K, McQueen S, Maller JJ, Herring S, Segrave R, et al. A randomized trial of rTMS targeted with MRI based neuro-navigation in treatment-resistant depression. Neuropsychopharmacology. 2009;34(5):1255-62. | 151 | 16.77 | 18 |
|  | Rumi DO, Gattaz WF, Rigonatti SP, Rosa MA, Fregni F, Rosa MO, et al. Transcranial magnetic stimulation accelerates the antidepressant effect of amitriptyline in severe depression: A double-blind placebo-controlled study. Biological Psychiatry. 2005;57(2):162-6. | 149 | 11.46 | 34 |
|  | Janicak PG, O'Reardon JP, Sampson SM, Husain MM, Lisanby SH, Rado JT, et al. Transcranial magnetic stimulation in the treatment of major depressive disorder: A comprehensive summary of safety experience from acute exposure, extended exposure, and during reintroduction treatment. | 148 | 14.80 | 21 |
|  | Eranti S, Mogg A, Pluck G, Landau S, Purvis R, Brown RG, et al. A randomized, controlled trial with 6-month follow-up of repetitive transcranial magnetic stimulation and electroconvulsive therapy for severe depression. American Journal of Psychiatry. 2007;164(1):73-81. | 147 | 13.36 | 25 |
|  | Padberg F, Zwanzger P, Keck ME, Kathmann N, Mikhaiel P, Ella R, et al. Repetitive transcranial magnetic stimulation (rTMS) in major depression: Relation between efficacy and stimulation intensity. Neuropsychopharmacology. 2002;27(4):638-45. | 145 | 9.06 | 43 |
|  | Martin JL, Barbanoj MJ, Schlaepfer TE, Clos S, Perez V, Kulisevsky J, et al. Transcranial magnetic stimulation for treating depression. Cochrane database of systematic reviews (Online). 2002(2). | 144 | 9.0 | 44 |
|  | Fregni F, Marcolin MA, Myczkowski M, Amiaz R, Hasey G, Rumi DO, et al. Predictors of antidepressant response in clinical trials of transcranial magnetic stimulation. International Journal of Neuropsychopharmacology. 2006;9(6):641-54. | 137 | 11.41 | 35 |
|  | Herwig U, Fallgatter AJ, HÃ¶ppner J, Eschweiler GW, Kron M, Hajak G, et al. Antidepressant effects of augmentative transcranial magnetic stimulation: Randomised multicentre trial. British Journal of Psychiatry. 2007;191(NOV.):441-8. | 127 | 11.54 | 33 |
|  | Jorge RE, Robinson RG, Tateno A, Narushima K, Acion L, Moser D, et al. Repetitive transcranial magnetic stimulation as treatment of poststroke depression: A preliminary study. Biological Psychiatry. 2004;55(4):398-405. | 127 | 9.07 | 42 |
|  | Eschweiler GW, Wegerer C, Schlotter W, Spandl C, Stevens A, Bartels M, et al. Left prefrontal activation predicts therapeutic effects of repetitive transcranial magnetic stimulation (rTMS) in major depression. Psychiatry Research - Neuroimaging. 2000;99(3):161-72. | 126 | 7.0 | 47 |
|  | Feinsod M, Kreinin B, Chistyakov A, Klein E. Preliminary evidence for a beneficial effect of low-frequency, repetitive transcranial magnetic stimulation in patients with major depression and schizophrenia. Depression and Anxiety. 1998;7(2):65-8. | 125 | 6.25 | 50 |
|  | Pridmore S, Bruno R, Turnier-Shea Y, Reid P, Rybak M. Comparison of unlimited numbers of rapid transcranial magnetic stimulation (rTMS) and ECT treatment sessions in major depressive episode. International Journal of Neuropsychopharmacology. 2000;3(2):129-34. | 124 | 6.88 | 48 |
